# Leadership tasks in public health: findings from the National Board of Public Health Examiners’ job task analysis

**DOI:** 10.3389/fpubh.2025.1583383

**Published:** 2025-06-16

**Authors:** Cerina Dubois, Anthony Dissen, Michael Bowen, Rick Kurz, Allison Foster, David Buys

**Affiliations:** ^1^Department of Mental Health, Bloomberg School of Public Health, Johns Hopkins University, Baltimore, MD, United States; ^2^School of Public Health, University of Alberta, Edmonton, AB, Canada; ^3^School of Health Sciences, Stockton University, Galloway, NJ, United States; ^4^Meazure Learning, Inc, Minneapolis, MN, United States; ^5^National Board of Public Health Examiners, Washington, DC, United States; ^6^Health Sciences and Biochemistry, Nutrition, & Health Promotion, Mississippi State University, Starkville, MS, United States

**Keywords:** public health, leadership, competency, job task analysis, work force

## Abstract

**Introduction:**

Public health leadership plays a crucial role in shaping effective health policies and practices. The National Board of Public Health Examiners (NBPHE) conducts a job task analysis (JTA) survey every 5–7 years to update the Certified in Public Health (CPH) examination. The objective of this study is to examine the JTA findings on leadership tasks in public health practice.

**Methods:**

In April 2022, through the collaboration of expert panels and a validation survey, 103 tasks organized into ten domains were established for the JTA survey. The JTA survey was distributed online to current public health professionals. Across the tasks in the ten domains, respondents were asked about frequency (Scale of 1–6; how often they performed this task) and criticality (Scale of 1–5; how important this task was to their job).

**Results:**

A total of 2,091 public health professionals responded to at least 82 of the 103 tasks (80%) and were included in the analysis. Approximately 86% of respondents worked in the United States and 41% had earned their CPH credential. Average frequency ratings ranged from 2.38 to 5.58, indicating that task ratings ranged from being performed never performed, every few years to daily. Average criticality ratings ranged from 2.46 to 4.64, indicating that task ratings ranged from not important to critically important. Specific to leadership, it was found that the ‘leadership’ domain ranked 2^nd^ highest for both frequency and criticality.

**Conclusion:**

Our findings suggest that leadership-focused development as part of academic public health programs and continuing education for the workforce is essential. Future research may examine how individuals perform on the leadership domain of the CPH exam across multiple characteristics to better inform additional workforce development strategies.

## 1 Introduction

Public health leadership plays a crucial role in shaping effective health policies and practices (1). Indeed, as the landscape of public health continues to evolve, the demand for skilled leaders who can navigate complex challenges and drive positive outcomes has never been greater. To work as an effective public health professional, one must possess both the skills and the dispositions needed to be an effective leader. Advocating for leadership skills and competence in the field of public health is crucial as it is the leaders who work to drive interventions, programs, and policies that address and improve the health of the public on a macro level (2). For example, during the beginning of the HIV and AIDS crises of the 1980s and beyond, it was those public health professionals who possessed strong leadership skills that were able to be a direct part of media advocacy, distribution of current research and evidence, and reduce fear, misinformation, and biases (3). This was more recently emphasized again during the COVID-19 pandemic, as public health leadership was a key component of delivering appropriate crisis management policies and crafting responses in real time (4).

Leadership is not synonymous with job rank or whether one possesses a job title at a managerial level. Leadership is something that can be found and demonstrated at all levels throughout an organization, regardless of one’s current level of authority or their position within a managerial bureaucracy or chain of command (4). To foster a leadership mentality within public health professionals, as well as to perform pre and post-professional education and training programs that are focused on leadership skills and dispositions, it is imperative to regularly assess what public health professionals are expected to do in the course of their work, especially as local, national, and global events shift and influence current and future public health needs. In doing so, it is possible to identify areas of strength, as well as areas of deficiency or gap, which can be used to reinforce or change curricular and professional development competencies in a real-time way. For example, the World Health Organization (WHO) has launched a public health competency framework that emphasizes key critical spots in relation to public health emergencies: flexibility, research, epidemiology, preparedness, and employability (5). Therefore, from a leadership perspective, new models are needed to prevent the siloing of professions and policies and instead form a more unified and synergistic approach to modern and future public health needs (6).

The field of public health has experienced changes related to the biological environment and in responses to ongoing infectious and chronic diseases (7). The COVID-19 pandemic is a primary example of these demands on public health with Mpox, bird flu, and similar viral outbreaks also occurring (8, 9). Additionally, public health professionals must continue to respond to chronic diseases such as cancer and heart disease through interventions that prevent their occurrence in a cost-effective manner, demonstrating the complex nature of supporting and promoting health and well-being within populations.

To investigate public health’s response to these and other critical demands, a better understanding of the work performed by the field is needed. One source of this information is the National Board of Public Health Examiners (NBPHE) job task analysis (JTA) survey conducted every 5–7 years to update the Certified in Public Health (CPH) examination, which is based on detailed information of the tasks and domains of public health work (10). The purpose of the CPH program is to professionalize the field of public health by demonstrating competence through certification. A JTA is a systematic process used to identify and document the specific tasks, responsibilities, and skills required for a particular job or practice, in this case, the public health profession (11). As public health professionals’ work changes over time, due to changes in disease risks, social determinants of health, and the outcomes of an ever-globalizing world, conducting a JTA ensures that CPH candidates are being tested on relevant tasks performed in the current field.

In 2022, the NBPHE conducted a comprehensive job task analysis study to identify the fundamental tasks and necessary knowledge for a Certified in Public Health (CPH) professional (12). Through the participation of subject matter experts and a survey distributed to current and former public health professionals, a content outline comprising ten domains and 95 specific tasks was developed and validated. Hence, this paper focuses on the results from the most recent iteration of the 2022 JTA study. The JTA was conducted to validate the domains and tasks identified by the panel using a larger sample of public health professionals. In this study, we will be focusing on the leadership domain and tasks in public health practice. We will explore two questions specific to the leadership domain and tasks.

1. How do the participants rank the frequency and criticality of their tasks in the leadership domain in comparison to the other domains?
2. What are the trends in comparing different roles within an organization regarding leadership tasks?

## 2 Materials and methods

### 2.1 Study design

In April 2022, through the collaboration of an expert panel and a validation survey, 103 tasks organized into ten domains were established for the JTA survey (10, 12). There were 3 phases to the development and implementation of the JTA survey which constitute the data for this study. Ethics approval by an institutional review board (IRB) was not required of this study as all human subject data were deidentified.

#### 2.1.1 Phase I: initial development

A panel of 18 subject matter experts assembled by the NBPHE gathered to create the test specifications for the JTA to accurately capture all the knowledge and skill areas within the current public health field and practice. The panel was comprised of employees from a variety of public health fields (to represent the range of domains in the content outline) including health departments, federal agencies, NGOs, and universities. Additional panelists were also selected to represent institutions outside of the United States.

Panelists were asked to assess the currency and relevancy of the current domains in the CPH exam content outline which were created through the initial NBPHE JTA in 2014 based on a factor analysis. They could add, edit, or remove the domains as needed. The panel determined that the public health profession could be divided into ten domains similar to those in the initial JTA:

- Data and Informatics
- Communication
- Leadership
- Law and Ethics
- Disease Prevention and Injury Reduction
- Community Engagement
- Program Planning and Evaluation
- Program and Resource Management
- Policy and Advocacy
- Health Equity and Social Justice

Panelists were then asked to review and revise the existing task statements from the previous JTA survey. The tasks underwent comprehensive revisions including keeping them as is, rewording of statements, being removed, or moved to more appropriate domains. In all, the panel’s work resulted in a final set of 103 tasks. Of these tasks, 18 were identified as part of the leadership domain, including:

1. Utilize evidence or data to inform decision-making and planning
2. Implement team-building skills and strategies to support and improve team performance
3. Motivate others within an organization or community to operate effectively
4. Establish and demonstrate standards of performance and accountability
5. Prioritize and justify allocation of resources
6. Encourage innovative solutions to current, persistent, and emerging problems
7. Apply conflict management skills
8. Develop strategies for collaborative and inclusive problem-solving, decision-making, and evaluation
9. Apply negotiation skills
10. Apply appropriate organizational change management concepts and skills
11. Communicate an organization’s or a community’s mission, goals, values, and shared vision to stakeholders
12. Develop capacity-building strategies at the individual, organizational, or community level
13. Contribute to the development, implementation, and evaluation of a strategic plan for an organization or with a community in conjunction with key stakeholders
14. Prepare professional development plans for self or others
15. Adapt organizational processes during times of crisis to enable business continuity
16. Evaluate organizational performance in relation to strategic and defined goals
17. Develop, implement, and evaluate a continuous quality improvement plan
18. Create teams for implementing community health initiatives

#### 2.1.2 Phase II: validation study

The items in the survey are the 103 job tasks. The JTA survey was distributed online to a large convenience sample of current public health professionals.

### 2.2 Survey design and measures

Using the domains and task statements identified by the JTA panel, Meazure Learning, an assessment and data analysis company, developed the JTA survey (13). The first section of the survey asked respondents to evaluate each task using the two scales: frequency and criticality.

- Frequency: “How often do you perform this task?” (Options of “1” meaning “Never” to “6” meaning “Daily”)
- Criticality: “How important is this task to you job?” (Options of “1” meaning “Not important” to “5” meaning “Critically important”)
- Average: The sum of frequency and criticality scores divided by 2.

The second section of the survey asked respondents to consider the 10 domains and provide the percentage of the examination that should be devoted to each domain. The final part of the survey asked for demographic information from the respondents to ensure a representative response and completion by appropriately qualified individuals.

The JTA survey link was sent both directly to public health professionals and to organizations representing public health professionals In addition, recipients were requested to forward the survey to relevant individuals within their respective organizations and academic institutions. The survey data was collected using Survey Monkey, a widely used online survey tool.

### 2.3 Reliability and validity

Reliability was assessed by Cronbach’s alpha, which was used to determine how consistently the survey covered the domains. Reliability was measured by internal consistency using Cronbach’s alpha on respondents’ ratings of frequency and criticality of each task. The reliability of the frequency and criticality ratings were 0.98 and 0.98, respectively, indicating near perfect agreement among respondents.

The validity of the tasks and domains was established by the initial expert panel review of the tasks and domains in use for the CPH exam, the perception of the respondents of the JTA survey, and a second review of the tasks and domains after the survey. This process resulted in a reduction in the number of tasks from 103 to 95 and the maintenance of the ten domains.

### 2.4 Statistical analysis

Summary descriptive statistics were captured from the data. These methods include measures of central tendency, such as the percentages, mean, median, and mode. Additionally, measures of dispersion, like range, variance, and standard deviation, indicate the spread or variability of the data. A one-way ANOVA was also conducted to determine any statistical differences across the 3 organizational levels entry, mid-level, and senior-level positions.

## 3 Results

2,091 complete (80% or more complete responses to the survey task items) responses were included in the analysis. The majority of the respondents were women (*n* = 1,425, 71.5%); and were ‘White’ (*n* = 1,163, 70.5%) (Table 1). Approximately 43% reported having mid-level positions; and approximately 86% of respondents worked in the United States and 41% had earned their CPH credential. For women respondents, the highest representation was observed in mid-level positions (33.4%), followed by senior-level (14.8%), entry-level (10.6%), and consultant (4.5%) positions.

**Table 1:** Demographics of the 2022 job task analysis survey respondents.

Characteristic	*N* (%)
Gender (*N* = 1,992)	
Men	516 (25.9)
Women	1,425 (71.5)
Other/prefer not to answer	51 (2.5)
Ethnicity (*N* = 1,649)	
American Indian/Alaska Native/Native Hawaiian/Pacific Islander	57 (3.5)
Asian	125 (7.6)
Black or African-American	207 (12.6)
White	1,163 (70.5)
Two or more races	97 (5.9)
Current role within organization (*N* = 1,985)	
Entry-level	276 (13.9)
Mid-level	860 (43.3)
Senior-level	465 (23.4)
Consultant	146 (7.4)
Clinical	115 (5.8)
Student	68 (3.4)
Other or did not answer	161 (8.1)
Employed in public health (*N* = 1,984)	
Yes	1,728 (87.1)
No	256 (12.9)

Specific to leadership, this domain scored 2nd highest for criticality and frequency (after the domain of ‘communication’) (Table 2). Likewise, it was found that the frequency and criticality of leadership tasks were rated above average for each organizational position level, though the frequency and criticality of leadership tasks were rated highest by senior-level respondents (Table 3). Notably, although average scores for ‘communication’ was higher than ‘leadership’, the top 15 tasks that ranked the highest overall (across all domains) fell in the ‘leadership’ domain. When scores were stratified by current role within organization levels, it was found that the ‘leadership’ domain ranked highest in all entry, mid, and senior-levels. Specifically, for senior-level respondents, 9 out of their top-scored 15 tasks were in the leadership domain, whereas mid-level was 5 out of 15, and entry was 4 out of 15 (Table 4). When comparing criticality results between the three groups (senior, mid-level, and entry), a one-way ANOVA demonstrated that the effect of level was significant for rating the criticality of leadership, *F* (2, 51) = 21.50, *p* < 0.001. Regarding frequency, one-way ANOVA demonstrated statistically significant difference in mean scores between groups in leadership frequency, *F* (2, 51) = 21.50, *p* < 0.001.

**Table 2:** Average, criticality, and frequency scores per domain.

Domain	Criticality	Frequency
Communication	3.8	4.1
Leadership	3.5	3.7
Community Engagement	3.4	3.5
Data and Informatics	3.3	3.5
Program Planning and Evaluation	3.3	3.3
Health Equity and Social Justice	3.3	3.3
Law and Ethics	3.2	3.3
Program and Resource Management	3.2	3.2
Disease Prevention and Injury Reduction	3.1	3.2
Policy and Advocacy	2.8	2.7

**Table 3:** Average overall job task analysis values across all domains stratified by current role in organization.

Domain	Senior-level	Mid-level	Entry-level
Leadership	3.95	3.50	3.21
Community Engagement	3.75	3.40	3.16
Communication	4.02	3.81	3.71
Program and Resource Management	3.77	3.12	3.72
Law and Ethics	3.50	3.17	2.99
Data and Informatics	3.59	3.36	3.18
Health Equity and Social Justice	3.48	3.23	3.11
Program Planning and Evaluation	3.50	3.29	3.00
Policy and Advocacy	3.24	2.59	2.38
Disease Prevention and Injury Reduction	3.35	2.86	2.85

**Table 4:** Domains of the top 15 ranked tasks by average of criticality and frequency scores stratified by current role in organization.

Domain	Senior-level	Mid-level	Entry-level
Leadership	9	5	4
Communication	4	4	5
Data and Informatics	–	2	2
Program and Resource Management	2	2	2
All other Domains	–	2	2
Total	15	15	15

Under the leadership domain, the task of ‘utilize evidence or data to inform decision-making and planning’ ranked the highest for average values, criticality, and frequency. Conversely, the task of ‘create teams for implementing community health initiatives’ scored lowest for average values, criticality, and frequency (Supplementary Tables 1–3).

## 4 Discussion

### 4.1 Ranking of leadership domain

This iteration of the JTA found that the domain of ‘leadership’ ranked 2^nd^ highest across all domains for average scores, criticality, and frequency. This suggests that leadership-focused development as part of academic public health programs and continuing education for the workforce is essential. This also affirms that the prioritization of leadership-focused task development in public health education and training programs will strengthen the effectiveness of the workforce and empower public health professionals to more effectively lead their teams, communities, and organizations to the requisite outcomes of the field.

### 4.2 Comparison to other literature

In comparison to other literature about leadership and public health, our study findings align with current literature that emphasizes leadership as a significant domain in public health professional practice (14, 15). Our approach to identifying the tasks performed by public health professionals is inductive and empirical asking for the perceptions of professionals in the field. Leadership, however, has been conceptualized in many ways, important among them is the distinction of transformational versus transitional leadership. Transformational leadership inspires and motivates individuals to perform beyond expectations regarding an organization’s vision and mission. Transactional leadership is based more on reinforcement and exchanges to get individuals to perform as expected and is often associated with the concept of management (16). This distinction can provide a basis for evaluating the tasks that public health professionals viewed as critical and occurring frequently. The conceptualization of this domain includes tasks related to both transformational and transactional characteristics of leadership (17). An overall review of the leadership tasks indicated (see list in the previous section) that both the transformational tasks (3) Motivate others within an organization or community to operate effectively (6), Encourage innovative solutions to current, persistent, and emerging problems (8), Develop strategies for collaborative and inclusive problem-solving, decision-making, and evaluation (10), Apply appropriate organizational change management concepts and skills (11), Communicate an organization’s or a community’s mission, goals, values, and shared vision to stakeholders (12), Develop capacity-building strategies at the individual, organizational, or community level and (13) Contribute to the development, implementation, and evaluation of a strategic plan for an organization or with a community in conjunction with key stakeholders and transactional tasks (1) Utilize evidence or data to inform decision-making and planning (2), Implement team-building skills and strategies to support and improve team performance (4), Establish and demonstrate standards of performance and accountability (5), Prioritize and justify allocation of resources (7), Apply conflict management skills (9), Apply negotiation skills (14), Prepare professional development plans for self or others (15), Adapt organizational processes during times of crisis to enable business continuity (16), Evaluate organizational performance in relation to strategic and defined goals (17), Develop, implement, and evaluate a continuous quality improvement plan, and (18) Create teams for implementing community health initiatives were identified by public health professionals who completed the validation survey, although most reflect the transactional management of existing personnel and resources. Although most of the critical, frequent and average scored tasks are transactional, survey respondents recognized that transformational tasks were also important to their work and needed to be performed with some frequency.

### 4.3 Application to recent events

Public health crisis illustrate the occurrence of both transactional and translational tasks. Particularly with the COVID-19 pandemic, this crisis presented a wide range of leadership challenges to public health professionals and organizations (18). Indeed, new reports have arisen from the pandemic on redefining leadership and how a range of leadership skills are critical in the new generation of public health leaders (1). As such, future JTAs should examine how individuals perform on the leadership domain of the CPH exam across multiple characteristics to better inform additional workforce development strategies.

### 4.4 Comparison across level of leadership roles

Particularly, our JTA found that there were significantly higher scores for ‘leadership’ by senior level respondents, in comparison to mid or entry levels (9 out of their top-scored 15 tasks were in the leadership domain). This aligns with competency frameworks in public health that emphasize the importance of senior public health professionals in leadership roles and the fact that senior professionals often take on more leadership-related tasks (15, 19). This is also supported by various theories and studies that report the influence of seniority on leadership responsibilities (20).

### 4.5 Future directions and the JTA

Conducting a job task analysis (JTA) that includes a leadership domain on public health professionals has important future implications for research, theory, and practice. By systematically identifying the essential tasks required for effective leadership, organizations can tailor training programs to better prepare current and future leaders. This ensures that public health leaders have the necessary skills to navigate complex health challenges, influence policy changes, and foster community trust. Additionally, the insights gained from a JTA can inform recruitment and professional development strategies, helping to build a more resilient and adaptive public health workforce.

From a theoretical perspective, the consistent data collected by the NBPHE through the JTA enhances the development and refinement of leadership models specific to public health. It builds upon the existing leadership framework for understanding how leadership competencies continue to evolve in response to emerging public health threats. This evolving framework can potentially act as a robust resource to inform both the theoretical and public health curriculum and education programs by providing direction on how the domain of leadership and its respective tasks change over time.

In terms of the application to future research, JTAs offer a rich, empirical foundation for longitudinal studies about the workforce that examine trends in their leadership roles, competencies and internal changes in public health over time. Researchers can utilize JTA data sets to explore correlations between leadership practices and public health outcomes, to evaluate the effectiveness of leadership training programs, and identify gaps in current educational programming. This data-driven approach led by the NBPHE will support public health workforce planning and leadership development.

Lastly, for practice, the application of the JTA findings may support public health organizations to identify gaps within leadership needs of the public health workforce. This includes updating the current training curriculum to reflect the most current and critical leadership tasks. This ensures that public health professionals are equipped with skills that are relevant to the evolving public health landscape. Notably, the integration of the JTA data into public health certification (such as the Certified in Public Health) and credentialing can help support standardization of leadership domains in public health.

Through continued surveillance of the public health workforce through JTAs and other similar processes, the NBPHE aims to become an open-access evidence-based resource center for all public health professionals, researchers, government workers, and academic institutions. This ongoing effort will strengthen the public health infrastructure and also foster a culture of continuous learning about the leadership domain at all levels of the workforce.

### 4.6 Strengths and limitations

The strength of this JTA survey is that it is one of its kind in the United States to specifically measure the domains and tasks of public health professionals. Typically used by the U.S. Office of Personnel Management, conducting these JTAs emphasizes the importance of establishing a standardized scope of practice surrounding public health professionals. This is the only JTA known to these researchers to specifically survey the domain of public health leadership in governmental public health workers.

However, the study is not without limitations. Although the sample of respondents is large and fairly representative of the public health workforce, it is not a random sample and hence, statistical inferences cannot be made. Also, the initial conceptualization of the domains and tasks were based on the work of a select group of subject matter experts; and thus, a different group may have produced a different outcome. Finally, the NBPHE has conducted two JTAs for the public health workforce. As the field evolves, different worker perceptions of the tasks that they are performing may affect the domains and tasks.

## 5 Conclusion

The findings of the 2022 JTA survey showed that the leadership domain and leadership-related skills, tasks, and competencies have been becoming increasingly important in the public health workforce. Continued JTAs are warranted to further understand which tasks and competencies are necessary to prepare current and future public health professionals, particularly those required for leadership positions.

## Data Availability

The raw data supporting the conclusions of this article will be made available by the authors, without undue reservation.
