# Supplementary material for: Leadership tasks in public health: findings from the National Board of Public Health Examiners’ job task analysis
Source: Front Public Health. 2025 Jun 16;13:1583383. doi: 10.3389/fpubh.2025.1583383 (PMC12206868; doi:10.3389/fpubh.2025.1583383)
Supplement: Supplementary file 1 [file Table_1.docx]

**Supplementary Tables**

**Supplementary Table 1. Leadership Domain Job Task Analysis Question Criticality Value by Rank**

| **Question** | **Senior-Level** | **Mid-Level** | **Entry-Level** |
| --- | --- | --- | --- |
| Utilize evidence or data to inform decision making and planning | 4.31 | 4.12 | 4.00 |
| Implement team building skills and strategies to support and improve team performance | 4.06 | 3.55 | 3.27 |
| Motivate others within an organization or community to operate effectively | 3.97 | 3.53 | 3.25 |
| Establish and demonstrate standards of performance and accountability | 4.00 | 3.82 | 3.57 |
| Prioritize and justify allocation of resources | 4.01 | 3.45 | 3.12 |
| Encourage innovative solutions to current, persistent, and emerging problems | 3.98 | 3.66 | 3.51 |
| Apply conflict management skills | 3.98 | 3.54 | 3.37 |
| Develop strategies for collaborative and inclusive problem solving, decision-making, and evaluation | 3.97 | 3.74 | 3.38 |
| Apply negotiation skills | 3.87 | 3.18 | 2.84 |
| Apply appropriate organizational change management concepts and skills | 3.75 | 3.13 | 2.73 |
| Communicate an organization’s or a community’s mission, goals, values, and shared vision to stakeholders | 3.76 | 3.33 | 3.24 |
| Develop capacity-building strategies at the individual, organizational, or community level | 3.69 | 3.12 | 2.91 |
| Contribute to the development, implementation, and evaluation of a strategic plan for an organization or with a community in conjunction with key stakeholders | 3.74 | 3.34 | 3.15 |
| Prepare professional development plans for self or others | 3.64 | 3.33 | 3.27 |
| Adapt organizational processes during times of crisis to enable business continuity | 3.68 | 3.06 | 2.72 |
| Evaluate organizational performance in relation to strategic and defined goals | 3.60 | 3.11 | 2.89 |
| Develop, implement, and evaluate a continuous quality improvement plan | 3.49 | 3.11 | 2.89 |
| Create teams for implementing community health initiatives | 3.31 | 2.72 | 2.61 |

**Supplementary Table 2. Leadership Domain Job Task Analysis Question Frequency Value by Rank**

| **Question** | **Senior-Level** | **Mid-Level** | **Entry-Level** |
| --- | --- | --- | --- |
| Utilize evidence or data to inform decision making and planning | 4.31 | 4.12 | 4.0 |
| Implement team building skills and strategies to support and improve team performance | 4.07 | 3.55 | 3.27 |
| Motivate others within an organization or community to operate effectively | 3.97 | 3.53 | 3.25 |
| Establish and demonstrate standards of performance and accountability | 4.00 | 3.82 | 3.57 |
| Prioritize and justify allocation of resources | 4.01 | 3.45 | 3.12 |
| Encourage innovative solutions to current, persistent, and emerging problems | 3.98 | 3.66 | 3.51 |
| Apply conflict management skills | 3.98 | 3.54 | 3.37 |
| Develop strategies for collaborative and inclusive problem solving, decision-making, and evaluation | 3.97 | 3.74 | 3.38 |
| Apply negotiation skills | 3.87 | 3.18 | 2.84 |
| Apply appropriate organizational change management concepts and skills | 3.75 | 3.14 | 2.73 |
| Communicate an organization’s or a community’s mission, goals, values, and shared vision to stakeholders | 3.76 | 3.33 | 3.24 |
| Develop capacity-building strategies at the individual, organizational, or community level | 3.69 | 3.12 | 2.91 |
| Contribute to the development, implementation, and evaluation of a strategic plan for an organization or with a community in conjunction with key stakeholders | 3.74 | 3.34 | 3.15 |
| Prepare professional development plans for self or others | 3.64 | 3.33 | 3.27 |
| Adapt organizational processes during times of crisis to enable business continuity | 3.68 | 3.06 | 2.72 |
| Evaluate organizational performance in relation to strategic and defined goals | 3.60 | 3.11 | 2.89 |
| Develop, implement, and evaluate a continuous quality improvement plan | 3.49 | 3.11 | 2.89 |
| Create teams for implementing community health initiatives | 3.49 | 3.11 | 2.89 |

**Supplementary Table 3. Leadership Domain Job Task Analysis Question Average Value by Rank**

| **Question** | **Senior-Level** | **Mid-Level** | **Entry-Level** |
| --- | --- | --- | --- |
| Utilize evidence or data to inform decision making and planning | 4.56 | 4.38 | 4.23 |
| Implement team building skills and strategies to support and improve team performance | 4.27 | 3.73 | 3.36 |
| Motivate others within an organization or community to operate effectively | 4.26 | 3.80 | 3.42 |
| Establish and demonstrate standards of performance and accountability | 4.21 | 4.09 | 3.78 |
| Prioritize and justify allocation of resources | 4.20 | 3.55 | 3.21 |
| Encourage innovative solutions to current, persistent, and emerging problems | 4.20 | 3.89 | 3.63 |
| Apply conflict management skills | 4.17 | 3.72 | 4.46 |
| Develop strategies for collaborative and inclusive problem solving, decision-making, and evaluation | 4.15 | 3.95 | 3.55 |
| Apply negotiation skills | 4.10 | 3.34 | 3.96 |
| Apply appropriate organizational change management concepts and skills | 3.92 | 3.25 | 2.78 |
| Communicate an organization’s or a community’s mission, goals, values, and shared vision to stakeholders | 3.84 | 3.43 | 3.25 |
| Develop capacity-building strategies at the individual, organizational, or community level | 3.76 | 3.18 | 2.92 |
| Contribute to the development, implementation, and evaluation of a strategic plan for an organization or with a community in conjunction with key stakeholders | 3.70 | 3.33 | 3.15 |
| Prepare professional development plans for self or others | 3.67 | 3.38 | 3.30 |
| Adapt organizational processes during times of crisis to enable business continuity | 3.66 | 3.03 | 2.64 |
| Evaluate organizational performance in relation to strategic and defined goals | 3.59 | 3.10 | 2.84 |
| Develop, implement, and evaluate a continuous quality improvement plan | 3.52 | 3.13 | 2.84 |
| Create teams for implementing community health initiatives | 3.30 | 2.70 | 2.53 |
